# Lessons learned from the use of 1,977 in-situ bilateral internal mammary arteries: a retrospective study

**DOI:** 10.1186/s13019-014-0158-9

**Published:** 2014-09-20

**Authors:** Siamak Mohammadi, Francois Dagenais, Pierre Voisine, Eric Dumont, Richard Baillot, Daniel Doyle, Eric Charbonneau, Dimitri Kalavrouziotis

**Affiliations:** Division of Cardiac Surgery, Quebec Heart and Lung Institute, Quebec City, QC Canada; Attending surgeon, Division of Cardiac Surgery, 2725 chemin Sainte-Foy, Quebec City, G1V 4G5 QC Canada

**Keywords:** Coronary artery bypass graft surgery, Arterial grafts, Outcomes

## Abstract

**Background:**

We sought to determine the early and long-term results of in-situ bilateral internal mammary artery (BIMA) grafting in patients undergoing coronary artery bypass graft surgery (CABG).

**Methods:**

Between 1992 and 2011, 16,364 patients underwent primary isolated CABG involving at least one in-situ IMA at our institution. Among these, 1,977 patients underwent in-situ BIMA grafting: the right IMA was used to revascularize the right coronary artery system in 1,279, the circumflex system in 454 patients, and the left anterior descending (LAD) in 244. Logistic and Cox regression analyses were used to predict in-hospital mortality and cumulative late death.

**Results:**

Late survival among BIMA patients was negatively and independently influenced by chronic obstructive pulmonary disease (hazard ratio (HR) 2.4, 95% confidence interval (CI) 1.6-3.4, *p =* 0.0005), age (HR 1.2, 95% CI 1.1-1.3, *p* < 0.001), and mediastinitis (HR 2.1, 95% CI 1.1-4.2, *p* < 0.03). Gender, body mass index, diabetes, choice of target for the second (non-LAD) IMA, and conduit grafted to the LAD (RIMA vs. LIMA) did not influence late survival among BIMA patients. A BIMA grafting strategy was significantly beneficial for younger patients. However, it was not associated with superior late survival for patients aged 66 years and above at the time of CABG, and showed a trend to harm among octogenarians (HR 1.05, 95% CI 0.70-1.56, *p* = 0.80).

**Conclusions:**

Female gender, non-insulin dependent diabetes, and the site of second IMA anastomosis did not influence early and long-term outcomes in patients undergoing CABG with in-situ BIMA grafting. The right and left IMAs are equally effective conduits for the LAD. However, advanced age, chronic obstructive pulmonary disease, and insulin-treated diabetes mellitus have a negative impact on late survival among patients with BIMA grafts.

**Electronic supplementary material:**

The online version of this article (doi:10.1186/s13019-014-0158-9) contains supplementary material, which is available to authorized users.

## Background

Given the superior late patency of the internal mammary artery (IMA) over venous grafts, it is reasonable to expect that bilateral internal mammary artery (BIMA) grafting would translate to enhanced survival following coronary artery bypass graft surgery (CABG). Numerous retrospective studies [1]-[3] have documented superior results associated with BIMA compared to single-IMA grafting. However, concerns about perioperative complications and the technical challenges inherent in BIMA grafting limit its broad utilization [4]. Our institution has previously reported that there may exist an age "cut-off" after which the survival benefit associated with BIMA grafting is lost [5]. However, limited power and a relatively short follow-up restricted our ability to identify the patient subgroups that would derive maximal benefit from BIMA grafting. Therefore, the objectives of the present study were two-fold: (1) to evaluate the late survival of an expanded number of a consecutive, real-world cohort of patients with BIMA grafting with longer follow-up and (2) to identify which patient characteristics, if any, make BIMA grafting prohibitive in patients undergoing CABG.

## Methods

### Patients

Clinical data were obtained from our computerized cardiac surgical database which collects information prospectively. Between January 1992 and February 2011, 16,364 consecutive adult patients underwent primary isolated CABG involving at least one in-situ IMA on the left anterior descending (LAD) at our institution. Among them, 1,977 patients with in-situ (proximally connected to the subclavian artery) BIMA grafts, with or without other supplemental venous conduits, were identified. The right IMA was used to revascularize the right coronary artery (RCA) system in 1,279, the circumflex system in 454, and the LAD in 244 patients. The left IMA was used to revascularize the LAD in 1,743, the circumflex system in 181, and the diagonal in 53 patients. The choice of the coronary territory receiving the IMAs was based on coronary anatomy and surgeon preference. All patients without a provincial health insurance number as well as those who had their CABG later in the study period (after 2009) were excluded from the long-term analysis. Therefore, long-term data were available for 1,862 patients (94.2%) with BIMA, and was 100% complete as of November 2009. The date of death was obtained from provincial vital statistics.

### Statistical analysis

Results are expressed as mean ± standard deviation (SD) or median as appropriate for continuous variables and proportions for categorical variables. Continuous and dichotomous variables were analysed using one-way ANOVA or Chi-square tests, respectively. Multivariate logistic regression was used to determine the predictors of in-hospital mortality as well as deep sternal wound infection (DSWI). Kaplan-Meier survival analysis was performed for late all-cause mortality and Cox proportional hazards multivariate regression modelling was used to estimate hazard ratios of late death among BIMA patients. Demographic, pre-, and intra-operative variables including territory grafted were presented to the model. Variables with a *p* < 0.25 on univariate analysis were candidates for model building. The adequacy of the proportional hazards assumption was verified using the graphical representation of the logarithm of the cumulative hazard rates versus time to assess parallelism and constant separation among the different values of nominal variables; the continuous variable age was stratified into 4 disjointed strata. Also, an artificially time-dependent covariate was added to the model to test the proportionality assumption. For all variables in the final model, proportional hazards assumptions were not rejected. Statistical significance was present when the two-tailed *p* value < 0.05. Analyses were performed using SAS version 9.2 (SAS Institute Inc., Cary, NC).

This study was in accordance with our institutional review board (Quebec Heart and Lung Institute Ethics Committee). Individual patient consent was waived due to the observational nature of the study.

## Results

### Patient characteristics

There were notable differences among BIMA and SIMA patients, with a significantly lower prevalence of several relevant risk factors in the former group (Table 1). The mean number of distal anastomoses was slightly higher among BIMA patients and aortic clamp and cardiopulmonary bypass (CPB) times were on average approximately 6 minutes longer in the BIMA cohort. The clinical characteristics of BIMA patients were further stratified by gender, body mass index (BMI), and diabetes (Table 2). There were 243 patients with diabetes (including 50 patients treated with insulin) among BIMA patients. Diabetic BIMA patients were on average 2 years older and had slightly higher BMI than non-diabetic BIMA patients; all other characteristics seemed to be equally distributed among diabetic and non-diabetic BIMA patients (Table 2). Among BIMA patients, there were 402 patients with a BMI > 30 kg/m^2^ (mean 32.6 ± 2.3); these patients were slightly younger than those with BMI ≤ 30 kg/m^2^ (Table 2). There were no major differences in the clinical characteristics of BIMA patients according to which IMA (right vs. left) was used to revascularize the LAD and the choice of non-LAD target receiving the second IMA (Table 3).

**Table 1:** **Baseline and operative characteristics**

Variable (% unless otherwise indicated)	BIMA	Single IMA	
	n = 1,977	n = 14,387	***p***value
Age (mean years ± SD)	55.5 ± 8.6	64.3 ± 9.6	<0.0001
Female	10.9	22.6	<0.0001
Body mass index (mean kg/m^2^ ± SD)	27.2 ± 3.7	27.9 ± 4.6	<0.0001
Diabetes mellitus	12.3	31.9	<0.0001
Insulin-dependent	2.5	9.7	<0.0001
Renal failure	3.4	7.4	<0.0001
Previous MI	50.5	52.3	0.13
Hypertension	49.3	64.0	<0.0001
Active smoking	32.2	22.0	<0.0001
COPD	6.2	13.1	<0.0001
Peripheral vascular disease	9.0	15.3	<0.0001
Previous stroke	3.1	6.3	<0.0001
LVEF ≤ 35%	3.9	6.7	<0.0001
CHF NYHA class III or IV	18.0	26.3	<0.0001
Atrial fibrillation	1.5	3.7	<0.0001
Three-vessel disease	58.7	53.0	<0.0001
Parsonnet score (mean ± SD)	1.4 ± 0.9	2.1 ± 2.2	<0.0001
EuroSCORE (mean ± SD)	1.9 ± 2.7	3.6 ± 4.5	<0.0001
*Operative Data*			
Urgent or emergency	18.5	23.1	<0.0001
Off-pump	2.3	3.2	0.03
Aortic clamp time (mean min ± SD)	57.6 ± 18.2	50.7 ± 18.3	<0.0001
CPB time (mean min ± SD)	82.4 ± 23.8	76.4 ± 23.7	<0.0001
Number of distals (mean ± SD)	3.6 ± 1.0	3.3 ± 1.0	<0.0001

*BIMA* bilateral internal mammary artery, *CPB* Cardiopulmonary bypass, *CHF* congestive heart failure, *COPD* chronic obstructive pulmonary disease, *IMA* internal mammary artery, *LVEF* left ventricular ejection fraction, *MI* myocardial infarction, *NYHA* New York Heart Association, *SD* standard deviation.

**Table 2:** **Baseline characteristics of BIMA patients stratified by gender, body mass index, and diabetes**

Variable (% unless otherwise indicated)	Male	Female		BMI > 30	BMI ≤ 30		Diabetes no insulin	No Diabetes	
	n = 1,761	n = 216	***p***value	n = 402	n = 1,575	***p***value	n = 193	n = 1,734	***p***value
Age (mean year ± SD)	55.0 ± 8.1	59.2 ± 11.5	<0.0001	54.3 ± 9.3	55.8 ± 8.4	0.002	57.4 ± 8.7	55.2 ± 8.5	0.0006
Female	---	---	---	11.2	10.9	0.86	9.3	10.8	0.62
BMI (mean kg/m^2^ ± SD)	27.3 ± 3.7	26.8 ± 4.1	0.09	32.6 ± 2.3	25.9 ± 2.6	<0.0001	28.7 ± 4.1	27.1 ± 3.6	<0.0001
Diabetes	12.2	13.4	0.59	18.7	10.7	<0.0001	---	---	---
Insulin-dependent	18.5	16.2	0.43	20.6	21.0	1.0	---	---	---
Renal failure	3.5	2.8	0.84	3.0	3.5	0.76	3.6	3.2	0.67
Previous MI	51.5	45.9	0.06	48.3	51.1	0.34	49.5	51.0	0.08
Hypertension	59.7	69.0	0.01	57.7	47.2	<0.001	65.8	68.9	0.15
Active smoking	32.4	30.7	0.64	29.3	32.4	0.12	28.3	32.5	0.06
COPD	6.4	4.6	0.37	6.0	6.3	0.9	7.3	6.1	0.53
Peripheral vascular disease	8.1	10.2	0.09	6.5	9.6	0.05	11.4	8.0	0.13
Previous stroke	2.9	5.1	0.09	3.2	3.1	0.87	2.6	3.2	0.83
LVEF ≤ 35%	3.8	4.8	0.45	3.6	4.0	0.88	4.2	3.6	0.68
CHF NYHA class III or IV	16.8	18.0	0.09	19.9	17.6	0.31	20.5	17.6	0.32
Atrial fibrillation	1.5	1.4	1.00	2.0	1.4	3.37	2.1	1.4	0.56
Three-vessel disease	59.2	54.6	0.21	59.2	58.6	0.86	62.7	58.2	0.25

*BIMA* bilateral internal mammary artery, *CPB* Cardiopulmonary bypass, *CHF* congestive heart failure, *COPD* chronic obstructive pulmonary disease, *IMA* internal mammary artery, *LVEF* left ventricular ejection fraction, *MI* myocardial infarction, *NYHA* New York Heart Association, *SD* standard deviation.

**Table 3:** **Baseline characteristics of BIMA patients stratified by use of left versus right IMA to revascularize the LAD (left part of table), and by choice of non-LAD target (RCA vs. circumflex) for the second IMA (right part of table)**

Variable (% unless otherwise indicated)	RIMA-LAD	LIMA-LAD	***p***value	2nd IMA-Cx	2nd IMA-RCA	***p***value
	n = 234	n = 1,743		n = 650	n = 1,239	
Age (mean years ± SD)	54.1 ± 8.7	55.6 ± 8.5	0.06	55.8 ± 8.4	55.4 ± 8.5	0.31
Female	10.8	11.0	0.9	10.7	11.1	0.88
BMI (mean kg/m^2^ ± SD)	27.3 ± 3.8	27.1 ± 3.7	0.83	27.3 ± 3.8	27.2 ± 3.6	0.65
Diabetes mellitus	13.8	12.2	0.49	13.7	12.5	0.52
Insulin-dependent	18.5	21.5	1.0	18.7	22.9	0.51
Renal failure	5.1	3.2	0.15	2.1	3.0	0.07
Previous MI	45.9	51.0	0.2	43.7	47.8	0.24
Hypertension	44.9	50.1	0.18	55.2	56.1	0.19
Active smoking	33.2	32.0	0.74	30.4	33.0	0.29
COPD	5.6	6.2	0.88	6.7	5.6	0.46
Peripheral vascular disease	8.2	8.9	0.79	9.9	8.5	0.34
Previous stroke	2.0	3.3	0.39	2.3	3.6	0.13
LVEF ≤ 35%	3.6	3.9	1.0	4.8	3.4	0.15
CHF NYHA class III or IV	19.5	18.0	0.62	15.5	17.3	0.55
Atrial fibrillation	2.6	1.4	0.22	1.5	1.3	0.83
Three-vessel disease	56.1	58.9	0.44	56.8	64.9	0.08
Parsonnet score (mean ± SD)	1.3 ± 0.9	1.4 ± 0.9	0.76	1.4 ± 1.0	1.3 ± 0.9	0.12
EuroSCORE (mean ± SD)	1.6 ± 1.4	1.9 ± 2.8	0.06	2.0 ± 2.9	1.9 ± 2.6	0.44

*BIMA* bilateral internal mammary artery, *CPB* Cardiopulmonary bypass, *CHF* congestive heart failure, *COPD* chronic obstructive pulmonary disease, *IMA* internal mammary artery, *LVEF* left ventricular ejection fraction, *MI* myocardial infarction, *NYHA* New York Heart Association, *SD* standard deviation.

### Early outcomes

#### In-hospital morbidity and mortality

The in-hospital mortality for the BIMA cohort was 1.0% (n = 20) compared with 1.8% (n = 264) among the single IMA patients (*p* = 0.009)(Table 4). The multivariate predictors of in-hospital mortality among the entire cohort of CABG patients with at least one in-situ IMA on the LAD were: age > 75 years (odds ratio (OR) 2.5, 95% confidence interval (CI) 1.8-3.4, *p* < 0.0001), female gender (OR 1.9, 95% CI 1.4-2.5, *p* < 0.0001), preoperative renal failure (OR 3.1, 95% CI 2.2-4.3, *p* < 0.0001), postoperative stroke (OR 4.3, 95% CI 2.7-6.8, *p* < 0.0001), postoperative de-novo renal failure (OR 3.1, 95% CI 2.2-4.2, *p* < 0.0001), mechanical ventilation > 48 hours (OR 11.2, 95% CI 8.0-15.6, *p* < 0.0001), and duration of CPB (OR 1.1, 95% CI 1.01-1.2, *p* < 0.0001). BIMA was not associated with in-hospital mortality on multivariate analysis (*p* = 0.43). Among BIMA patients, the site of the second IMA anastomosis to a non-LAD target (*p* = 0.33), the use of the left versus right IMA to revascularize the LAD (*p* = 0.25), female gender (*p* = 0.47), and DSWI (*p* = 0.39) were not associated with in-hospital mortality on univariate logistic regression.

**Table 4:** **Postoperative adverse events**

Outcome (% unless otherwise indicated)	BIMA	Single IMA	
	n = 1,977	n = 14,387	***p***value
Mortality	1.0	1.8	0.009
De-novo atrial fibrillation	15.6	23.3	<0.0001
Re-exploration for bleeding	3.7	3.7	0.96
De-novo renal failure*	3.9	7.0	<0.0001
Stroke	0.8	1.7	0.003
Deep sternal wound infection	2.4	1.2	<0.0001
Mechanical ventilation > 48 hours	1.8	2.8	0.01
Blood product transfusion	42.2	48.5	<0.0001
Mean hospital length of stay (days)	5	6	0.01

*Defined as a 50-μmol/L absolute rise in baseline serum creatinine or a new need for dialysis.

*BIMA* bilateral internal mammary arteries.

#### Deep sternal wound infection

The incidence of DSWI was doubled in the BIMA patients (2.4% (n = 48) vs. 1.2% (n = 172), *p* < 0.0001)(Table 4). Diabetes was more prevalent among patients who developed DSWI compared to those who did not have this complication (37.5% vs. 11.7%, *p* < 0.0001). Mean age (58.2 ± 8.1 vs. 55.4 ± 8.6, *p* = 0.02), mean BMI (29.8 ± 4.3 vs. 27.2 ± 3.7, *p* < 0.0001), mean Parsonnet score (8.0 ± 5.9 vs. 4.9 ± 4.9, *p* < 0.0001) and the prevalence of chronic obstructive pulmonary disease (COPD)(14.6% vs. 6.0%, *p* = 0.02), peripheral vascular disease (20.8% vs. 8.7%, *p* = 0.01), and female gender (20.8% vs. 10.7%, *p* = 0.03) were higher among BIMA patients who developed DSWI compared to those who did not. The multivariate independent predictors of DSWI in the entire cohort are shown in Table 5. In this model, no statistically significant effect modification was identified between BIMA and the other predictors of DSWI.

**Table 5:** **Multivariate predictors of deep sternal wound infection**

Variable	Odds ratio	95% CI	***p***value
BIMA	3.8	2.6-5.4	<0.0001
Diabetes mellitus	2.2	1.6-2.9	<0.0001
COPD	2.2	1.6-3.1	<0.0001
Preoperative dialysis-dependent renal failure	3.1	1.3-7.9	0.01
Postoperative delirium	1.6	1.04-2.4	0.03
Postoperative renal failure*	2.2	1.5-3.2	<0.0001
Reexploration for bleeding	2.6	1.6-4.2	<0.0001
Mechanical ventilation > 48 hours	4.6	3.0-7.0	<0.0001
Body mass index^†^	1.12	1.1-1.2	<0.0001
Left ventricular ejection fraction^†^	0.9	0.8-0.92	<0.0001

*Defined as a 50-μmol/L absolute rise in baseline serum creatinine or a new need for dialysis.

^†^Submitted to the model as continuous variables.

*CI* confidence interval, *CPB* cardiopulmonary bypass.

#### Long-term survival

**Figure 1:**
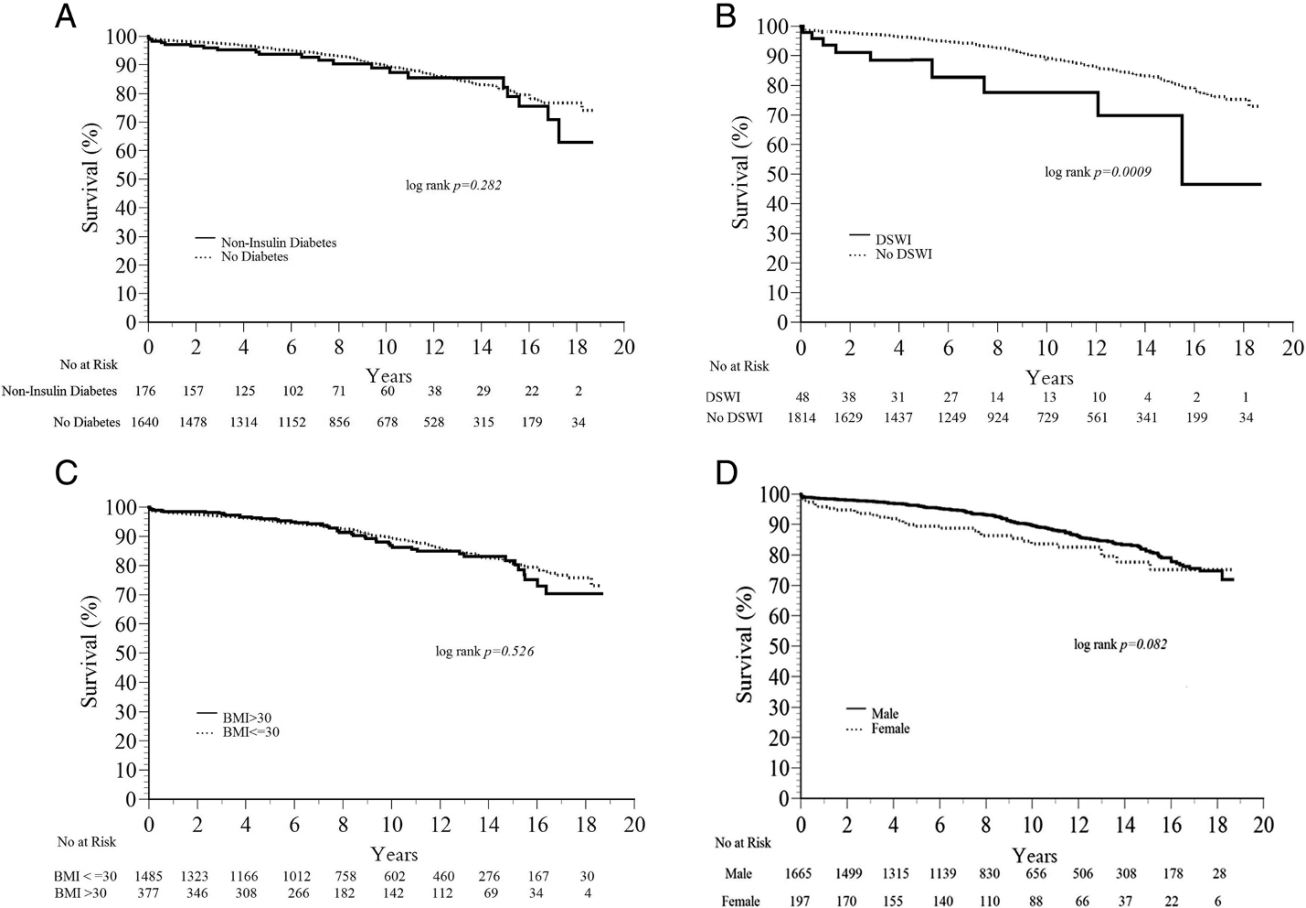
**Survival among BIMA patients comparing: A) Non-insulin dependent diabetes and patients without diabetes; B) Patients who developed DSWI versus those who did not; C) Patients with BMI > 30 kg/m** ^**2**^ **versus patients with BMI ≤ 30 kg/m** ^**2**^ **; D) Female versus male.**

The median follow-up of BIMA patients was 8.1 years (interquartile range, 4.7 to 13 years). During follow-up, there were a total of 208 deaths in the cohort of patients with BIMA. BIMA patients with diabetes had significantly lower unadjusted survival rates (91.7%, 84.4% and 77.0%) compared to BIMA patients without diabetes at 5, 10, and 15 years (96.0%, 89.8% and 81.5%, log-rank *p =* 0.006). However, once insulin-dependent diabetics were removed from this analysis, there was no longer a survival disparity between BIMA patients without diabetes and those with non-insulin dependent diabetes (log-rank *p* = 0.28, Figure 1A). BIMA patients with DSWI had lower 5-, 10-, and 15-year survival (85.6%, 77.6% and 69.8%) compared to BIMA patients without this type of infection (95.8%, 89.4% and 81.2%, log-rank *p =* 0.0009, Figure 1B). There were no significant late survival differences among BIMA patients stratified by BMI (log-rank *p* = 0.53) and gender (log-rank *p* = 0.08) (Figure 1C, D).

**Figure 2:**
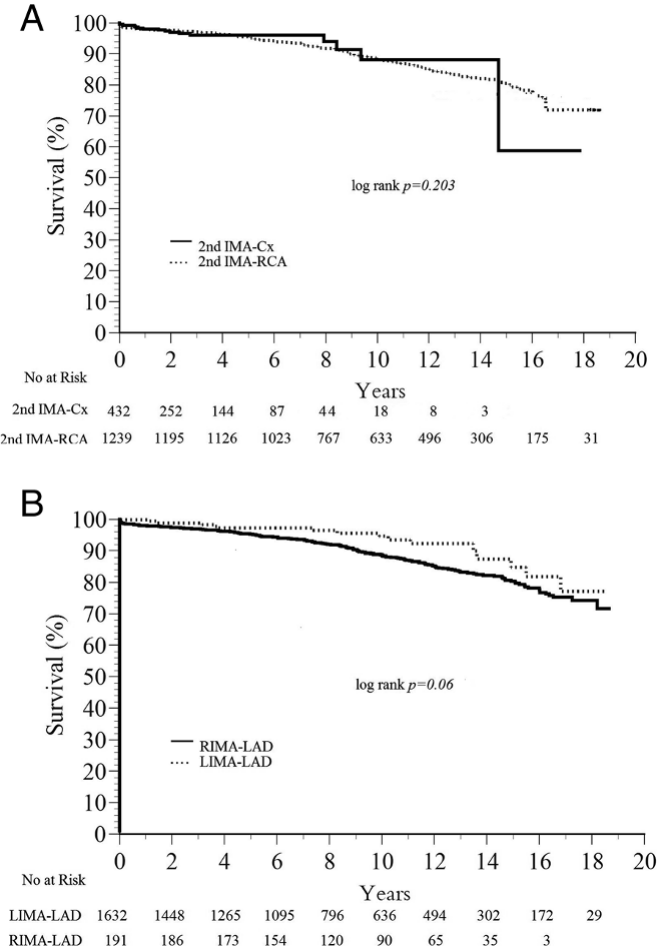
**Survival among BIMA patients based on A) Site of anastomosis of the second (non-LAD) IMA; B) Use of the left versus right IMA to revascularize the LAD.**

When we looked at the non-LAD target of the second IMA, unadjusted survival rates at 5, 10, and 15 years were 95.6%, 89.0% and 80.9% for patients with a second IMA anastomosis to the RCA system, which was not significantly different compared to those in whom the second IMA was anastomosed to the circumflex system (96.0%, 92.1% and 84.0%, log-rank *p =* 0.2*,* Figure 2A). Furthermore, using the left versus the right IMA to revascularize the LAD had no impact on late survival, although there was a trend toward better survival among those patients who received LIMA to the LAD (log-rank *p =* 0.06*,* Figure 2B).

The Cox multivariate proportional hazards model identified age, COPD, and DSWI as the independent predictors of late death among BIMA patients (hazard ratio (HR) 1.2, 95% CI 1.1-1.3, *p* < 0.001; HR 2.4, 95% CI 1.6-3.4, *p =* 0.0005; and HR 2.1, 95% CI 1.1-4.2, *p* < 0.03, respectively). The site of the second (non-LAD) IMA anastomosis, the use of a sequential technique, the use of the right versus the left IMA to bypass the LAD, female gender, BMI, and non-insulin dependent diabetes were not associated with long-term mortality among BIMA patients in this model. Analysis stratified by age shows that survival benefit is lost after 65 years of age, and that there may be a survival detriment associated with BIMA at age 80 (Table 6).

**Table 6:** **Hazard ratios of late mortality according to age among BIMA patients**

Age (years)	Hazard Ratio (95% CI)	***P-***value
50	0.64 (0.51-0.82)	0.0003
55	0.70 (0.58-0.84)	0.0001
60	0.76 (0.65-0.89)	0.0007
65	0.82 (0.68-0.99)	0.0412
66	0.83 (0.69-1.01)	0.0759
67	0.85 (0.6-1.04)	0.1272
68	0.86 (0.6-1.07)	0.1953
69	0.88 (0.70-1.10)	0.2779
70	0.89 (0.70-1.14)	0.3711
75	0.97 (0.70-1.56)	0.8550
80	1.05 (0.70-1.56)	0.7978

For clarity, age categories are shown at 5-year intervals except between the ages of 65 and 70 where they are shown per annum, in order to determine the age between 65 and 70 years at which the benefit of BIMA is no longer statistically significant. This occurs at 66 years of age.

## Discussion

Several findings in this study are worthy of note. First, a very low operative mortality (1%) was observed in this contemporary, real-world cohort of BIMA patients. These data are consistent with a recent randomized trial examining BIMA vs. single IMA which reported a 30-day mortality of 1.2% [6]. Second, the choice of target for the second (non-LAD) IMA did not influence late survival. Previous reports had suggested that the patency of the right IMA may be lower if placed to the RCA territory due to size discrepancy and competitive flow [7],[8]. More recent data from the Cleveland Clinic group [9] and others [10] have shown that the right IMA could be placed to left-sided coronaries or, alternatively, RCA territory targets with similar early and late outcomes. Although we observed a trend to an improved unadjusted survival among BIMA patients in which the LIMA was grafted to the LAD compared to those in which the RIMA was grafted to the LAD (log-rank *p* = 0.06, Figure 2B), this factor was not predictive of late survival in the Cox multivariate model.

Another relevant finding from our data is that the use of insulin was the major driver behind the survival disadvantage of diabetes in patients undergoing BIMA, such that there was no difference in survival among diabetics who were not receiving chronic outpatient insulin therapy and those without diabetes (Figure 1A). Looking at the data from Table 2, we see that there a very few clinically relevant differences between the patients with diabetes not treated with insulin receiving BIMA and those without diabetes receiving BIMA which may explain the equivalent survival outcome. The contribution of diabetes to adverse events and mortality following BIMA grafting remains controversial with conflicting results in the literature [11]-[14]. However, our data suggest that diabetes in the absence of insulin therapy should not deter a surgeon from pursuing a BIMA revascularization strategy.

The fear of sternal devascularisation and infection is a major limitation in the widespread use of BIMA. However, recent advances in the management of DSWI have lessened its impact on CABG outcomes [13]. The role of diabetes as a major risk factor for DSWI has been extensively studied. The prevalence of diabetes was three times higher among patients with DSWI versus those without DSWI in our series which is consistent with observations in the Arterial Revascularisation Trial (ART) [6]. In an earlier report, Cosgrove et al. [14] studied the risk of BIMA grafting and identified diabetes and advanced age, but not BIMA grafting itself, as risk factors for wound complications. Although the incidence of DSWI after BIMA grafting is relatively low in most series, the morbidity and cost associated with sternal infection are significant [12],[15]. This increase in the risk of sternal infection in diabetic patients has to be balanced against the fact that diabetic patients may actually have the most to gain from BIMA grafting [16],[17]. Our data show that, excluding diabetics treated with insulin, diabetic BIMA patients had a significantly better survival compared to single IMA patients. The presence of end-organ compromise related to diabetes, associated risk factors, and IMA harvesting technique should be taken into consideration for surgical decision-making [18]. The risk of DSWI may be minimized with careful patient selection and modification of the IMA harvesting technique, particularly in diabetic patients. Furthermore, in our study, diabetes was not an independent predictor of late death after BIMA grafting. A recent report by our group [19] has also shown that long-term survival after CABG was not adversely affected by the presence of non-insulin-dependent diabetes. These findings suggest that long-term survival of diabetic patients after CABG may be mainly related to the presence of diabetes co-morbidities, namely peripheral vascular disease, renal failure, low ejection fraction, and insulin dependency. Stevens et al. [17] have shown that BIMA use in diabetic patients improves survival and decreases the risk of coronary reoperation compared with single IMA grafting.

Another finding worthy of note is that female gender, patients with high BMI, and history of smoking did not negatively influence long-term survival after BIMA grafting in our study. The finding of a lack of association between BMI and survival according to grafting strategy may reflect a lack of sufficient power in our study to detect a difference, given the fact that BMI was extremely similar among the 2 groups (Table 1) and very obese patients (BMI > 40) were conspicuously absent from the BIMA group. Earlier studies have shown that female gender is an independent risk factor for in-hospital and long-term mortality after CABG. It has been also suggested that women derive less benefit from BIMA grafting. However, recent reports [20],[21] have not validated these data, and women appear to benefit from BIMA grafting as much as men.

### Limitations

This study has some inherent limitations. It was performed in a non-randomized manner in a single referral center. Also, our analysis does not account for the time-dependent nature of certain patient characteristics, such as ejection fraction and diabetes, which are prognostically important and may change over time. The prevention of graft disease has evolved with the introduction of new drug therapies during follow-up, but we expect these new treatments to have been equally distributed among the groups we studied. Our data suggest that the late survival benefit appears to be lost after the age of 65 for patients undergoing BIMA grafts. It is not unreasonable to expect that these patients may continue to experience sustained improvements in anginal status and quality of life owing to better long-term patency of arterial grafts; however, these variables were not specifically assessed in this study. We excluded free IMA grafts and alternative arterial conduits such as the radial artery from our analysis in order to increase the homogeneity of the studied patients and allow for meaningful multivariate analyses within this patient subgroup.

## Conclusion

An in-situ BIMA grafting strategy should not be withheld from patients according to sex, BMI, and from those with diabetes mellitus. However, advanced age, COPD, and patients with insulin-treated diabetes mellitus may not benefit from BIMA grafts. The right and left IMAs are equally effective conduits for the LAD, and the non-LAD coronary artery territory grafted by the second IMA did not influence outcome.

## Authors’ original submitted files for images

Below are the links to the authors’ original submitted files for images. Authors’ original file for figure 1 Authors’ original file for figure 2 Authors’ original file for figure 3 Authors’ original file for figure 4 Authors’ original file for figure 5 Authors’ original file for figure 6

## Abbreviations

CABG: Coronary artery bypass graft surgery
CI: Confidence interval
COPD: Chronic obstructive pulmonary disease
CPB: Cardiopulmonary bypass
BIMA: Bilateral internal mammary arteries
BMI: Body mass index
DSWI: Deep sternal wound infection
HR: Hazard ratio
IMA: Internal mammary artery
LAD: Left anterior descending artery
LIMA: Left internal mammary artery
OR: Odds ratio
RCA: Right coronary artery
SD: Standard deviation
