# Supplementary figures and images for: Lessons learned from the use of 1,977 in-situ bilateral internal mammary arteries: a retrospective study
Source: J Cardiothorac Surg. 2014 Sep 20;9:158. doi: 10.1186/s13019-014-0158-9 (PMC4177259; doi:10.1186/s13019-014-0158-9)

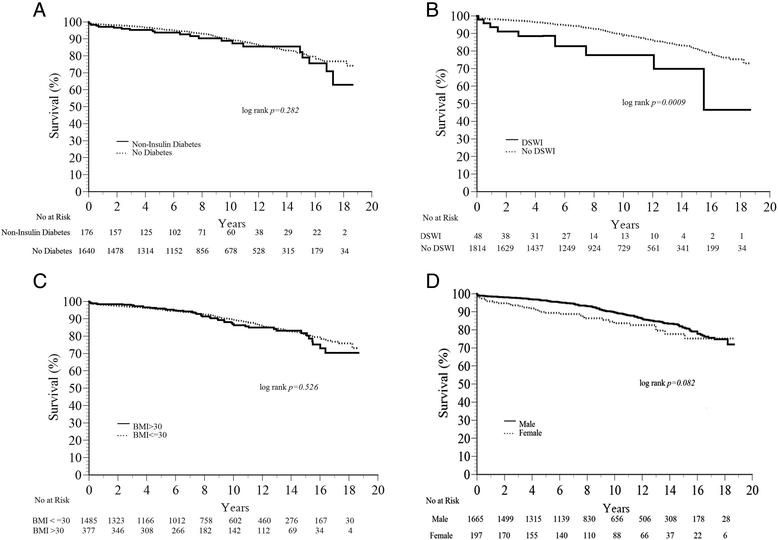

Supplement: Supplementary file 1 — Authors’ original file for figure 1 [file 13019_2014_158_MOESM1_ESM.gif]

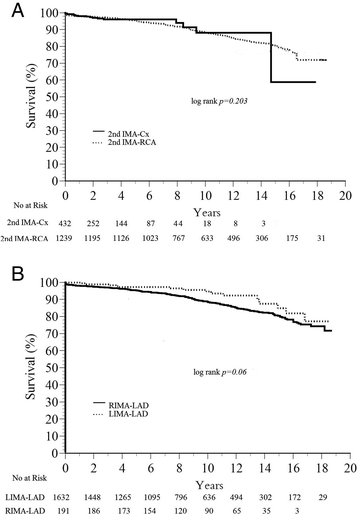

Supplement: Supplementary file 2 — Authors’ original file for figure 2 [file 13019_2014_158_MOESM2_ESM.gif]

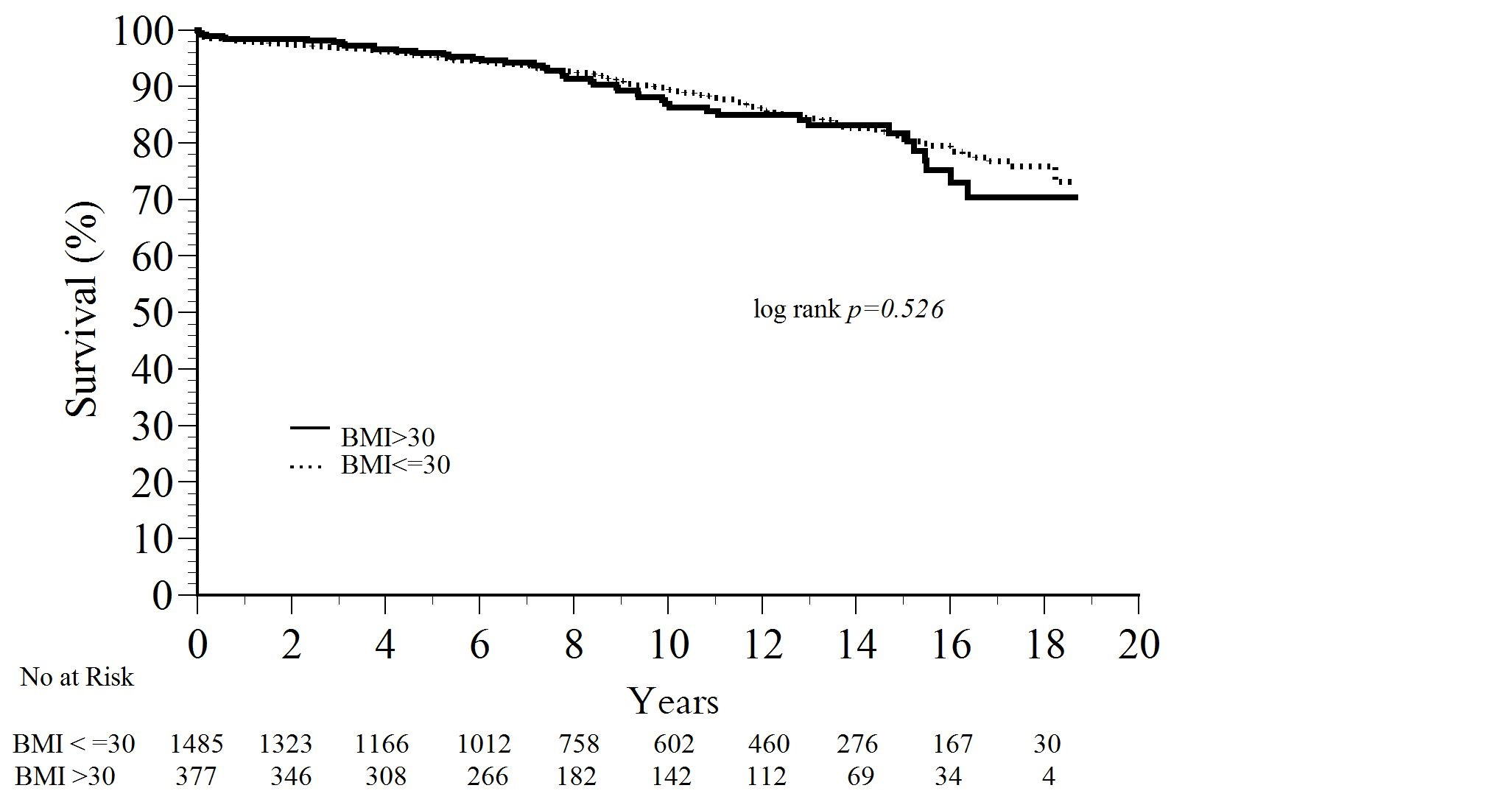

Supplement: Supplementary file 3 — Authors’ original file for figure 3 [file 13019_2014_158_MOESM3_ESM.tiff]

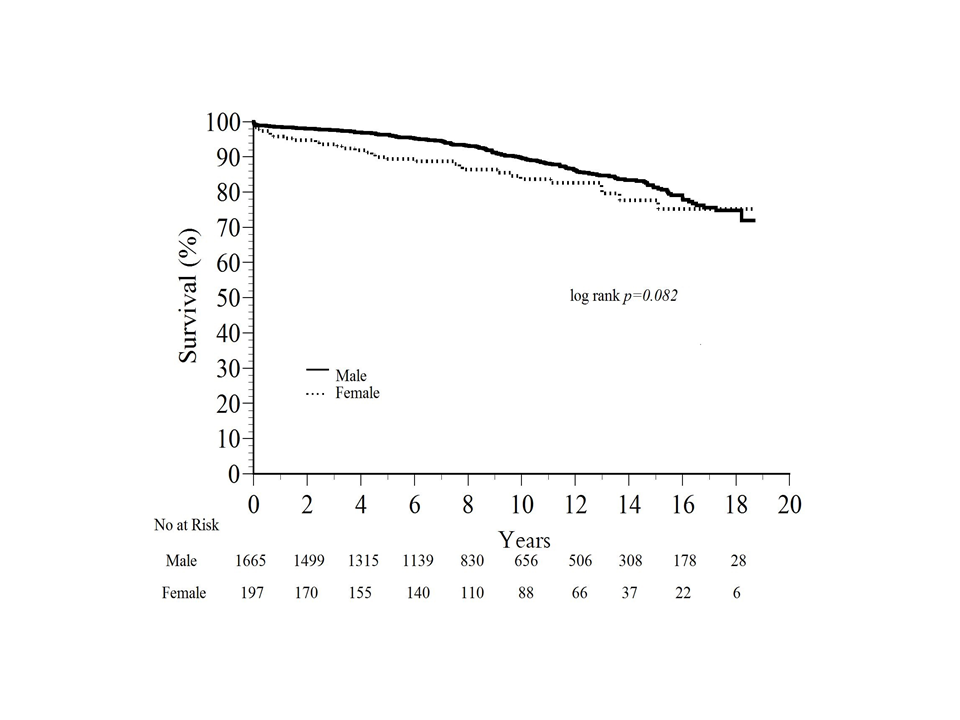

Supplement: Supplementary file 4 — Authors’ original file for figure 4 [file 13019_2014_158_MOESM4_ESM.tiff]

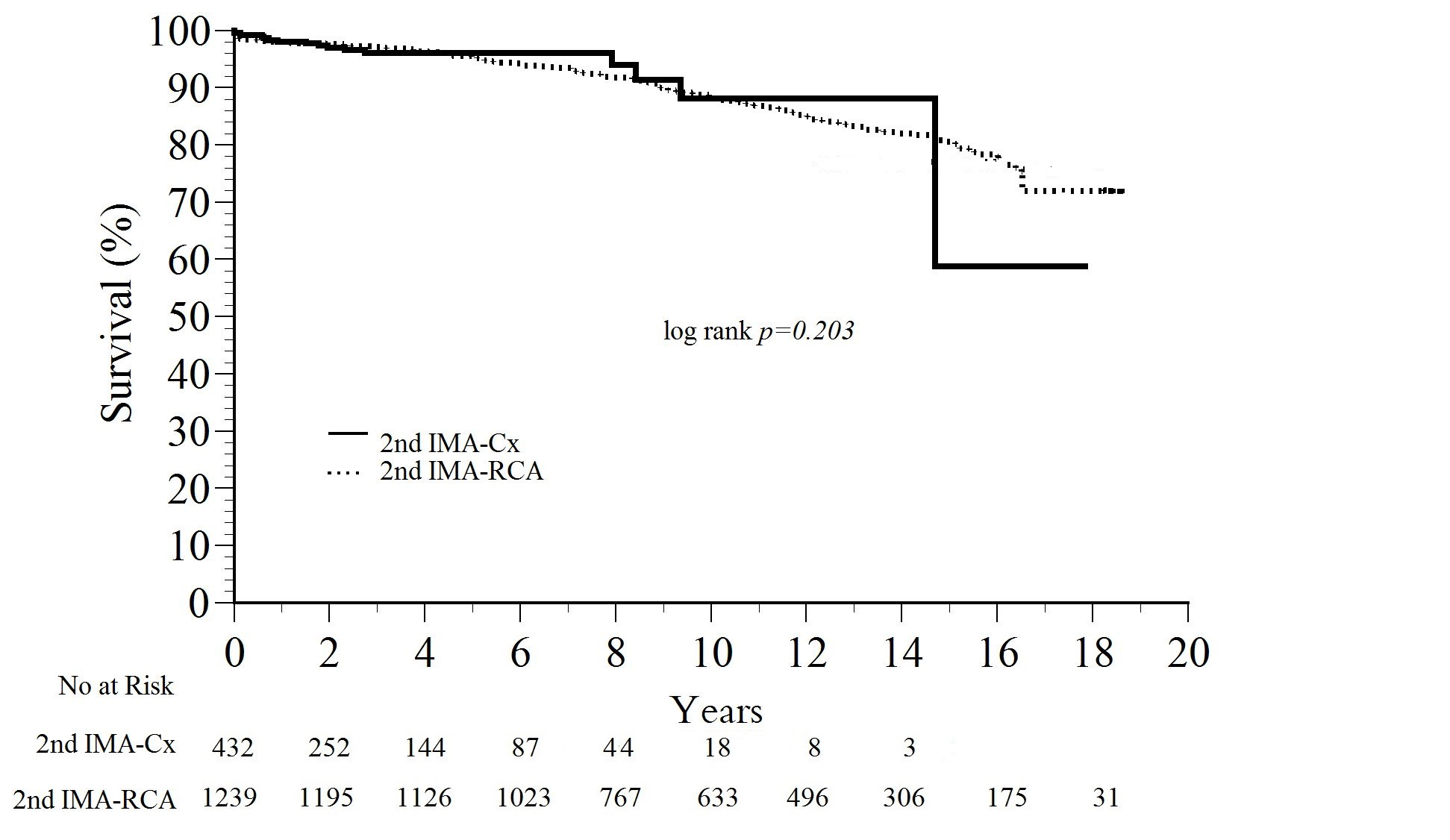

Supplement: Supplementary file 5 — Authors’ original file for figure 5 [file 13019_2014_158_MOESM5_ESM.tiff]

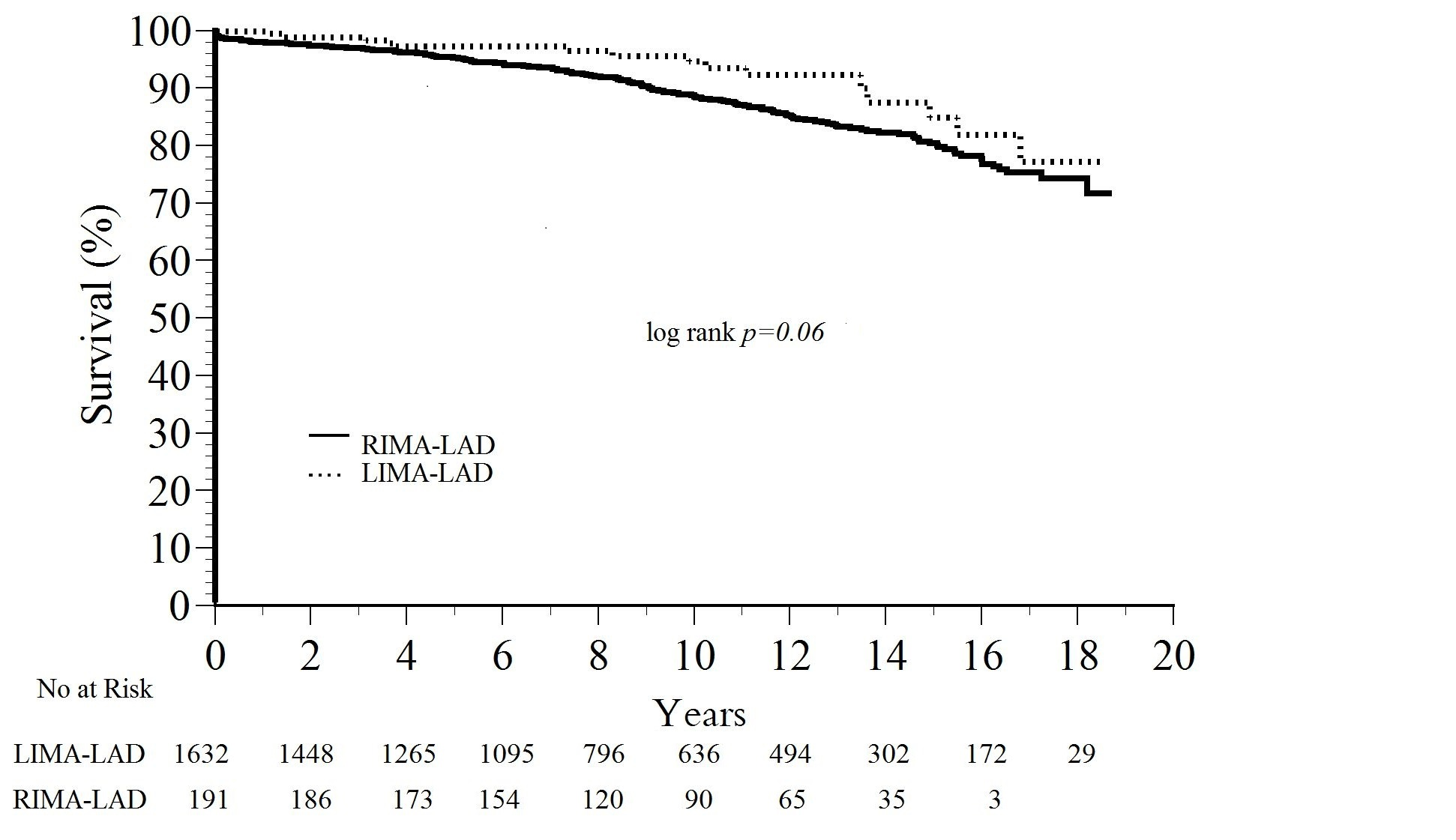

Supplement: Supplementary file 6 — Authors’ original file for figure 6 [file 13019_2014_158_MOESM6_ESM.tiff]
